# The effect of clamped and unclamped umbilical cord samples on blood gas analysis

**DOI:** 10.1007/s00404-021-06076-w

**Published:** 2021-05-22

**Authors:** Elisabetta Colciago, Simona Fumagalli, Elena Ciarmoli, Laura Antolini, Antonella Nespoli, Salvatore Andrea Mastrolia, Paolo Emilio Tagliabue, Chiara Furlan, Cristina Manganini, Patrizia Vergani

**Affiliations:** 1 Department of Medicine and Surgery, University of Milano-Bicocca, Milan, Italy; 2 San gerardo Hospital, Monza, Italy; 3 Neonatal Intensive Care Unit, Fondazione MBBM, San Gerardo Hospital, Monza, Italy; 4 Department of Obstetrics and Gynecology, Ospedale dei Bambini “Vittore Buzzi”, University of Milano, Milan, Italy; 5 Fondazione MBBM, Monza, Italy

**Keywords:** Blood gas analysis, Umbilical cord, Acid–base equilibrium, Cord clamping

## Abstract

**Purpose:**

Delayed cord clamping for at least 60 s is recommended to improve neonatal outcomes. The aim of this study is to evaluate whether there are differences in cord BGA between samples collected after double clamping the cord or without clamping the cord, when blood collection occurs within 60 s from birth in both groups.

**Methods:**

A cross-sectional study was carried out, collecting data from 6884 high-risk women who were divided into two groups based on the method of cord sampling (clamped vs unclamped).

**Results:**

There were significant decrease in pH and BE values into unclamped group compared with the clamped group. This difference remained significant when considering pathological blood gas analysis parameters, with a higher percentage of pathological pH or BE values in the unclamped group.

**Conclusion:**

Samples from the unclamped cord alter the acid–base parameters compared to collection from the clamped cord; however, this difference does not appear to be of clinical relevance. Findings could be due to the large sample size, which allowed to achieve a high power and to investigate very small numerical changes between groups, leading to a statistically significant difference in pH and BE between samples even when we could not appreciate any clinical relevant difference of pH or BE between groups. When blood gas analysis is indicated, the priority should be given to the timing of blood collection to allow reliable results, to assess newborns status at birth and intervene when needed.

## Introduction

The acid–base status in umbilical cord arterial blood at birth reflects the newborn’s aerobic and anaerobic intrauterine metabolisms and is an objective retrospective measure of the fetal well-being during labour [1–3].

One of the four criteria for defining an intrapartum hypoxic-ischemic event that could lead to cerebral palsy, is the presence of acidosis (pH ≤ 7.00 and/or BE ≤ − 12 mmol/L) at birth [4].

The umbilical cord arterial blood gas analysis (cABG) should be performed to identify if a fetal hypoxia/acidosis occurred [5]. To adhere to the recommendation guidelines suggesting to delay cord clamping for at least 60 s after a baby is born, to find the best technique to collect the umbilical cord arterial blood sample at birth without altering the cABG appears crucial.

The standard technique for obtaining umbilical cord arterial blood should be to double clamp the cord at birth and to collect the sample from the intervening segment [6], to allow paired cord blood gases to be taken [7].

Evidence suggest that sampling of cord arterial blood for gas analysis may be performed on the unclamped cord immediately after birth [8], allowing placental transfusion to provide additional blood to the newborn [9]. This could be a safe strategy to collect umbilical blood without altering the cABG values [10] as already demonstrated in a low-risk population [11–14].

Regardless of the technique, sample should be collected as soon as possible following birth, to avoid alteration of the cord arterial blood gas analysis, due to their values in umbilical blood that are quickly changing after delivery [3, 5, 15, 16].

Given that the international guidelines [10–12] recommend to perform a cABG only when an antepartum or intrapartum complication occurred or when the baby is in poor condition at birth, it is important to identify only the high-risk population who would benefit from the cABG. Moreover, an appropriate technique should be adopted, to have a reliable cABG and to allow a delayed cord clamping. In light of the lack of evidence available on this issue, we conducted a retrospective study in a high-risk population to ascertain whether there are differences in umbilical cord arterial blood gas analysis cABG between blood samples collected with different techniques: after double clamping the cord or on the unclamped cord, taking both samples within 60 s from birth.

## Materials and methods

A cross-sectional study was carried out. The study was conducted in a Consultant-led Unit with approximately 2700 births per year. Data were collected from the birth register between 1st January 2013 and 31st December 2017.

Within this unit, the recommended technique for umbilical cord arterial blood sampling between 1st January 2013 and 30th June 2015 was to double clamp the umbilical cord immediately after birth (within 60 s from birth) and to collect the blood sample from the double clamped cord section. Since 1st July 2015, the recommended technique was to perform a delayed cord clamping, collecting the blood from the unclamped pulsating cord within 60 s from birth, to allow sampling from the umbilical artery and, at the same time, placental transfusion. After sample collection, the midwife was placing a finger over the punctured site to avoid blood loss. The umbilical cord could be double clamped within 3 min from birth. Blood samples collected between 1st January 2013 and 30th June 2015 were labelled as Group A, while those collected between 1st July 2015 and 31st December 2017 were labelled as Group B.

Blood collection was obtained, for both groups, using heparinate syringes and was analysed immediately after birth using an automatic blood gas analyser. Arterial blood sample was analysed for pH and base excess (BE) and values were compared between the two groups.

The target population was represented by high-risk women with maternal or fetal complications during pregnancy. Women with multiple pregnancy or preterm birth (the obstetric unit has dedicated protocols on cord blood collection for these populations), cases with maternal (placental abruption, sepsis) or fetal (requiring immediate resuscitation at birth) intrapartum complications that would change the procedure of cord blood collection, arterial blood gas samples for which pH, BE or both were not available, where excluded from the study.

Within the study the pH and BE values were defined as pathological when < 7.00 and ≥ − 12 mmol/L, respectively, according to the FIGO and the ACOG recommendations [10–12].

### Statistical analysis

Descriptive analysis of maternal characteristics, maternal and fetal complications, and intrapartum variables was obtained by means and standard deviations (continuous variables), and by percentages (categorical variables). Distribution of continuous variables was compared across both groups using *T* test. Chi-square test was adopted for the comparison of categorical variables. A two sided 5% significance level was used for testing.

Confidence intervals on difference between theoretical means and on single theoretical means were calculated by *T* asymptotic approximation. Confidence intervals on difference between proportions and on single proportions were calculated by Gaussian asymptotic approximation. A 95% confidence level was used for confidence intervals on differences between parameters among groups. A 97.5% confidence level was used for confidence intervals on single theoretical parameters within groups, to account for multiplicity.

A multivariable linear regression model and a logistic regression model were performed to relate the pH and BE continuous variables or binary variables to both blood cord collection techniques, adjusting for potential confounders.

### Ethical approval

Authors and data retrieval assistants attended “Good Clinical Practice” training on ethical and organizational standards. The study has been performed in accordance with the ethical standards laid down in the 1964 Declaration of Helsinki and its later amendments. The present study was exempt from IRB approval as per Institutional policy on retrospective studies. At our medical center, women provide a written consent to the use of their clinical anonymized and de-identified data upon admission.

## Results

**Fig.1:**
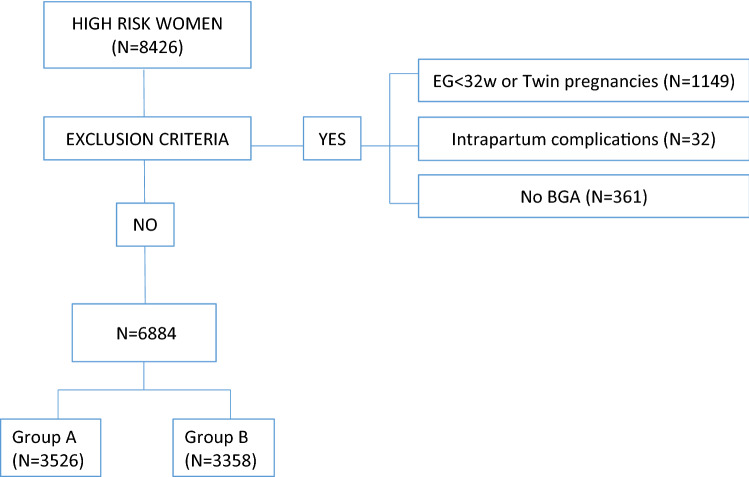
Flow chart sample size

A total of 8426 high-risk women who gave birth between 1st January 2013 and 31st December 2017 were screened for the eligibility criteria (Fig. 1). A number of 6884 women who fulfilled the criteria and were recruited for the study. They were categorized based on the year of birth which matched with the clamped cord group (Group A = 3526) or with the unclamped cord group (Group B = 3358).

Maternal characteristics, maternal and fetal clinical characteristics, and intrapartum complications of both groups are reported in Table 1. A significant difference between groups was found for BMI values (*P* = 0.018), previous uterine surgery (*P* < 0.001), other maternal complications (*P* < 0.001), macrosomia (*P* < 0.001), polyhydramnios (*P* < 0.001), spontaneous onset of labour *(P* < 0.003), induction of labour *(P* < 0.005) and epidural analgesia (*P* < 0.046).

**Table 1:** Description of the study groups and clinical variables

		Overall (*n* = 6884)		Group A (*n* = 3526)		Group B (*n* = 3358)		*p* value
		Mean (*n*)	SD (%)	Mean (*n*	SD (%)	Mean (*n*	SD (%)	
Maternal characteristics	Maternal age (years)	33.3	5.5	33.2	5.5	33.5	5.4	0.069
	BMI	23.9	4.9	23.8	4.7	24.1	5.1	0.018
	Gestational Age (weeks)	39	1.89	38.98	1.93	39.03	1.85	0.282
	Parity (primiparous)	4352	63.2	2238	63.5	2114	63.0	0.656
Maternal clinical characteristics	Diabetes	1194	17.34	600	17.02	594	17.69	0.461
	Ipertensive disorders	491	7.13	260	7.37	231	6.88	0.425
	Thyroid disorders	856	12.43	418	11.85	438	13.04	0.135
	Previous uterine surgery	1301	18.90	512	14.52	789	23.50	0.001
	Other maternal complications*	367	5.33	235	6.66	132	3.93	0.001
Pregnancy complications	Macrosomia	187	2.72	131	3.72	56	1.67	0.001
	IUGR	424	6.16	198	5.62	226	6.73	0.054
	Maformation	180	2.61	104	2.95	76	2.26	0.074
	Olygohydramnios	379	5.51	207	5.87	172	5.12	0.173
	Polyhydramnios	504	7.37	308	8.74	196	5.84	0.001
	Placental abnormalities ^#^	117	1.70	61	1.73	56	1.67	0.841
Intrapartum variables	Onset of labour							
	Spontaneous	2928	42.53	1561	44.27	1367	40.71	0.003
	Induction of labour	2711	39.38	1332	37.78	1379	41.07	0.005
	No labour	1245	18.09	633	17.95	612	18.23	0.769
	Epidural analgesia	2068	36.7	1025	35.4	1043	38.0	0.046
	Mode of birth Vaginal	4644	82.4	2384	82.4	2260	82.3	0.784
	Caesarean	1967	28.57	1011	28.67	956	28.47	0.852
	Vacuum assisted	273	3.97	131	3.72	142	4.23	0.275

*BMI* body mass index, *IUGR* intra uterine growth restriction, *SD* standard deviation

*Other maternal complications = heart diseases, lung disease, Kidney disease, autoimmune disease, metabolic disease, coagulopathy

^#^Placental abnormalities = Placenta praevia, accreta, percreta, increta

**Fig. 2:**
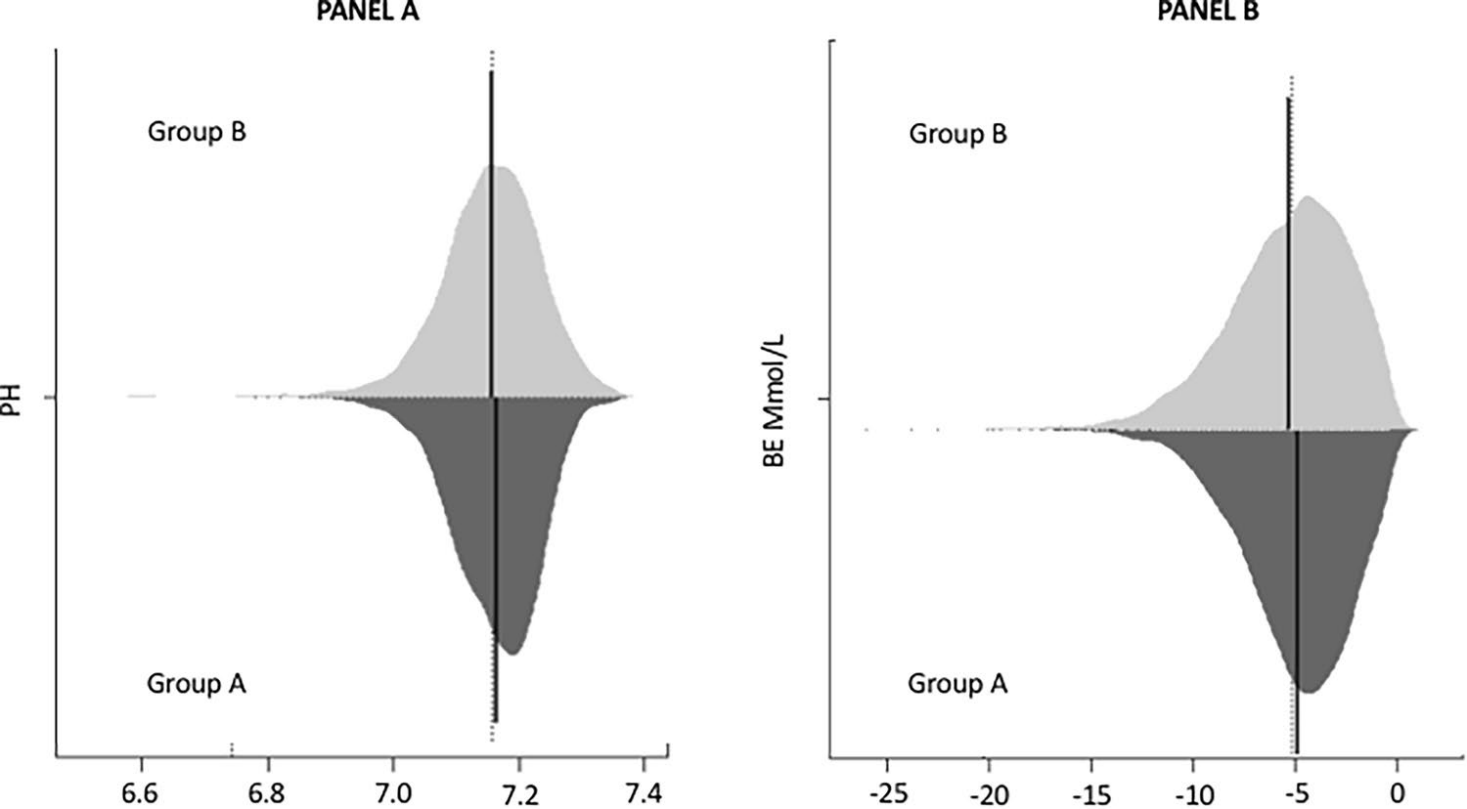
Bean Plot: Distribution of pH (panel A) and BE (panel B) into two groups clamped (group A) and unclamped (group B)

Figure 2 shows the distribution of the primary outcome of both pH (panel A) and BE (panel B) values into Group A and Group B. The two distributions have been compared using a Bean Plot. Means of pH and BE values between the two groups were significantly different (*P* < 0.0001 for both) as reported in Table 2. The average pH value was 7.26 in Group A and 7.25 in Group B, with a 98% CI of 7.259–7.263 and 7.251–7.257, respectively. The average BE value was − 4.53 in group A and − 4.97 in group B, with a CI of − 4.612 to − 4.439 Mmol/L and − 5.073 to − 4.864 Mmol/L, respectively. Group B showed a higher percentage of abnormal pH values compared to the percentage found into Group A (0.68% vs. 0.31%, respectively) (Table 2). The same was found for BE values with 2.44% pathological BE into group B and 1.13% pathological BE into Group A (Table 2).

**Table 2:** Umbilical cord blood gas analysis divided into Group A and Group B

	Overall (*n* = 6884)		Group A (*n* = 3526)		Group B (*n* = 3358)		*p* value
	Mean (*n*)	SD (%)	Mean (*n*)	SD (%)	Mean (*n*)	SD (%)	
pH	7.26	0.08	7.26	0.07	7.25	0.08	0.0001
BE	− 4.74	2.86	− 4.53	2.62	− 4.97	3.08	< 0.001
Pathological pH	34	0.49	11	0.31	23	0.68	0.027
Pathological BE	122	1.77	40	1.13	82	2.44	0.000
Pathological BGA*	125	1.82	42	1.19	83	2.47	0.000

*BE* base excess, *BGA* blood gas analysis

*pH < 7 or BE ≥ − 12 mmol/L

The 97.5% confidence interval for the proportions of pH pathological values in Group A were CI 0.14–0.61% and in Group B were CI 0.40–1.09%. The 97.5% confidence interval for the proportions of BE pathological values in Group A were CI 0.76–1.62% and in group B were CI 1.86–3.14%. The percentages of pathological cABG were significantly different between the two groups (*P* < 0.001), with a percentage of normal cABG of 98.8% and 97.5%, respectively.

In the multivariable linear regression model showed in Table 3, adjusted for confounders, the coefficient β on the contrast between Group B and Group A is − 0.0076. This represents the difference between the expected value of pH in a newborn into group B and the expected value of pH in a newborn into group A, where these neonates have common levels of the other variables included in the regression model. Of note, the coefficient β was very similar to the difference between the average values observed in both groups (Table 2). The same concept can be applied for the multivariable linear regression model showed in Table 3 for the BE. To improve the interpretation of findings we performed a power analysis. The sample size of both groups achieved 90% power to reject the null hypothesis of equal theoretical means of the pH and the BE, when the absolute value of the difference between the means of the two groups was 0.078 standard deviations. According to this, assuming a standard deviation equal to the maximum one observed, which are 0.08 for the pH and 3.08 for the BE, the minimum numbers needed to obtain a differences statistically significant between the two groups were 0.063 for the pH and 0.240 Mmol/L for the BE.

**Table 3:** Regression coefficients: effect of clamped and unclamped umbilical cord samples on pH and BE values, adjusted for maternal-foetal characteristics and intrapartum variables

Variable	Umbilical cord BGA (*n* = 6884)					
	pH			BE		
	β	(95% CI)	*p* value	β	(95% CI)	*p* value
Group B (vs group A)	− 0.0076	(− 0.0113; − 0.0038)	0.001	− 0.4454	(− 0.5789; − 0.3120)	0.001
BMI	− 0.0006	(− 0.0010; − 0.0003)	0.001	− 0.0032	(− 0.0167; 0.0103)	0.644
Other maternal disease*	0.0089	(0.0007; 0.0171)	0.033	0.3381	(0.0452; 0.6312)	0.024
Fetal polyhydramnios	− 0.0013	(− 0.0083; 0.0058)	0.726	− 0.1744	(− 0.4262; 0.0774)	0.175
Fetal macrosomia	− 0.0074	(− 0.0188; 0.0040)	0.203	− 0.0772	(− 0.4857; 0.3314)	0.711
Spontaneous labour	− 0.0275	(− 0.0329; − 0.0221)	0.001	− 2.3016	(− 2.4953; − 2.1079)	0.001
Induction of labour	− 0.0309	(− 0.0369; − 0.0249)	0.001	− 2.5002	(− 2.7148; − 2.2856)	0.001
Epidural analgesia	− 0.0096	(− 0.0140; − 0.0052)	0.001	− 0.0805	(− 0.2380; 0.0770)	0.317
Previous uterine surgery	0.0048	(− 0.0002; 0.0098)	0.062	0.3493	(0.1699; 0.5287)	0.001

*BGA* blood gas analysis, *BMI* body mass index, *BE* base excess

*Other maternal complications = heart diseases, lung disease, Kidney disease, autoimmune disease, metabolic disease, coagulopathy

In the multivariable logistic regression model considering the presence of pathological cABG adjusted for the unbalance factors among the two groups, the coefficient on the contrast between Group B and Group A gave an OR = 2.16 with a 95% CI 1.43–3.25.

## Discussion

As suggested from other authors [10] cord umbilical arterial blood can be taken from a pulsating and an unclamped umbilical cord without altering gas analysis results; therefore, we started to collect blood from the pulsating and unclamped cord, to allow placental transfusion and to improve neonatal outcomes [15]. After more than two years that this practice has been introduced, with this study we aimed to ascertain whether in a high-risk population there are differences in umbilical cord cABG between samples collected with clamped and unclamped cord, both within 60 s from birth.

This is the first study considering a large sample size with more than 6000 women in a 5 years’ time, with the aim to observe the effect of blood cord collection technique on the umbilical cord arterial blood gas analysis (cABG) in a high-risk population. Findings showed a statistically significant difference in pH and BE between the clamped and the unclamped group. Ackerman [3] was the first to demonstrate a significant change in pH and pCO_2_ when sampling was performed in infants within 60 s from birth. Our results are in agreement with this and with findings reported in other researches [1–3], observing a trend towards an acidosis in the unclamped cohort, although we did not take into account other than pH and BE parameters. When a pathological cABG was considered (pH < 7 or BE ≤ − 12 Mmol/L) our study showed a significant difference between groups, with a higher percentage of pathological pH and BE in the unclamped cord blood samples.

In contrast with other authors [10], our findings suggested that in a high-risk population pH and BE values are sensitive to the sampling procedure. For the interpretation of findings, our large sample size needs to be considered. The differences reported could be due to the large sample size, that allowed to achieve a high power even when we could not appreciate any clinical relevant difference of pH or BE between groups. The population size allowed to investigate very small numerical changes between groups, leading to a statistically significant difference in pH and BE between samples collected on the unclamped or the clamped cord. This could also explain why the difference observed between groups had no clinical importance.

In accordance with previous findings [12, 15, 17] the BE values showed the most critical changes [12]. As already demonstrated by other authors [17, 18], these alterations could be time-dependant; umbilical cord arterial blood gas analysis parameters decrease when cord sampling is not immediately performed, showing even a further drop when collection occurs between 45 and 90 s from birth [19]. We are not aware about the exact time of sampling in our cohort, and can only report that blood has been collected within 60 s from birth, as per protocol. Therefore, it appears that in a high-risk population the priority should be given to the timing of blood collection, to perform a reliable cABG, and not to the clamping technique [19].

This observation reinforces another crucial consideration already investigated [19] that umbilical cord arterial blood gas analysis might be influenced by the onset of newborn’s breathing. Even after spontaneous breathing, newborns have poor oxygenation which increases when the ventilation becomes more established. When this occurs, the baby starts to eliminate CO_2_ from the lungs and acids are released into the blood, leading to a decrease in BE values. In our study, newborns’ breathing varied considerably due to the large sample size, we, therefore, could confirm that BE values are dependent from the onset of newborns’ breathing.

The large sample size could also explain the difference between groups among maternal and neonatal complications in pregnancy; however, they were not statistically different when considering the pathological cABG in the multivariable regression model.

Although the observed pH and BE changes were of no clinical relevance, findings showed that newborns in the unclamped group were more likely to present an acidosis, this means that further evidence considering a high-risk population are needed. In fact, the advantages of both immediate umbilical cord arterial blood sampling for acid–base assessment and delayed cord clamping, should be evaluated, especially for these newborns who are the ones who could benefit even more from the placental transfusion effect [9, 10, 12, 17, 20].

When there is a clinical indication to perform the cABG, cord blood should be taken through a technique which allows reliable results, to assess newborns status at birth and intervene when needed.

## Strengths and limitations of the study

First of all, the strength of this study lies in the large size of the study sample. Another advantage of this study is the robust multivariable generalized estimating equations performed. The study is not a randomized trial, population differences may still be considered due to chance as data were retrospectively collected. Another limitation could be due to the historic comparison between the two groups.

## Conclusion

Blood collection from an unclamped cord is a safe option, allowing for placental transfusion benefits also within a high-risk population. Blood taken from the pulsating and unclamped cord group, showed no clinical relevant changes in pH and BE values. However, newborns in the unclamped group were more likely to present an acidosis, this means that a randomized clinical trial should be conducted in a high-risk population, to strengthen our results.
